# Gold Nanomaterials and Bone/Cartilage Tissue Engineering: Biomedical Applications and Molecular Mechanisms

**DOI:** 10.3389/fchem.2021.724188

**Published:** 2021-07-09

**Authors:** Yifeng Shi, Xuyao Han, Shuang Pan, Yuhao Wu, Yuhan Jiang, Jinghao Lin, Yihuang Chen, Haiming Jin

**Affiliations:** ^1^ Department of Orthopaedics, The Second Affiliated Hospital and Yuying Children’s Hospital of Wenzhou Medical University, Wenzhou, China; ^2^ College of Chemistry and Materials Engineering, Wenzhou University, Wenzhou, China; ^3^ The Second School of Medicine, Wenzhou Medical University, Wenzhou, China

**Keywords:** gold nanomaterials, bone/cartilage, tissue engineering, molecular mechanism, uptake

## Abstract

Recently, as our population increasingly ages with more pressure on bone and cartilage diseases, bone/cartilage tissue engineering (TE) have emerged as a potential alternative therapeutic technique accompanied by the rapid development of materials science and engineering. The key part to fulfill the goal of reconstructing impaired or damaged tissues lies in the rational design and synthesis of therapeutic agents in TE. Gold nanomaterials, especially gold nanoparticles (AuNPs), have shown the fascinating feasibility to treat a wide variety of diseases due to their excellent characteristics such as easy synthesis, controllable size, specific surface plasmon resonance and superior biocompatibility. Therefore, the comprehensive applications of gold nanomaterials in bone and cartilage TE have attracted enormous attention. This review will focus on the biomedical applications and molecular mechanism of gold nanomaterials in bone and cartilage TE. In addition, the types and cellular uptake process of gold nanomaterials are highlighted. Finally, the current challenges and future directions are indicated.

## Introduction

Bone and cartilage play a vital role in providing mechanical support, mobility and weight bearing for the body. At present, millions of patients are suffering from bone and cartilage degeneration and diseases, such as fractures, osteoporosis, low back pain and osteoarthritis (Zhang and Jordan, 2010; Agarwal and Garcia, 2015). Bone and cartilage-related medical treatments and costs are rising with the increase in life expectancy of the population. Therefore, tissue engineering (TE) is gradually considered a potential alternative therapeutic technique that could provide regeneration platform for bone and cartilage tissue loss or damage.

Commonly, TE involves the proliferation, stimulation, differentiation, and guidance of cells with the goal of reconstructing impaired or damaged tissues. There are three critical factors in a successful tissue regeneration: cells, scaffolds, and signaling mediators (e.g., growth factors) (Li H. et al., 2020). The repair of bone and cartilage tissue is a complicated event involving cells, signal molecules and suitable scaffolds to prepare new tissue in special environment (Keeney et al., 2011). Furthermore, the rapid development of materials science and engineering has promoted the progress of alternative medical methods for bone and cartilage diseases. Bone and cartilage TE along with modern nanomaterials science has made a significant contribution to health care and will expand further with the increasing elderly population.

Nanomaterials are tiny particles with size distribution less than 100 nm. In the last decades, various types of nanomaterials have attracted the attention of many researchers in photocatalysis (Pan et al., 2021), electrocatalysis (Zhang et al., 2021a), photoelectrocatalysis (Hu et al., 2020; Zhao et al., 2020; Huang et al., 2021), solar utilization (Jiang et al., 2020), heat management (Zhang et al., 2021b) and other fields because of their unique properties (Wang et al., 2019; He et al., 2020). Among the numerous materials, Au represents one well-studied type (Xu et al., 2016) with tunable shape (Zhu et al., 2015), structure (Ma et al., 2015), and composition (Jiang et al., 2017). For gold nanomaterials, AuNPs, as well as nanoclusters (AuNCs), nanocages, nanorods (AuNRs), nanobelts, nanoplates and so on, are becoming increasingly popular because of their optical nonlinearities (Gao et al., 2015; Huang et al., 2017; Zhang et al., 2018), local surface plasmon resonance (SPR) (Li Q. et al., 2017) and photothermal effect (Jiang et al., 2012). Considering these characteristics, it has been proved that gold nanomaterials can be used in many fields such as chemistry, biomedicine, including the diagnosis and treatment of diseases (Li et al., 2018b), sensor (Li et al., 2015), catalysis (Li et al., 2013), surface-enhanced Raman spectroscopy (Huang et al., 2019), illumination (Zhang et al., 2017b), detector (Du et al., 2018), and therapy (Yeh et al., 2012). The easy of synthesis and the unique properties of gold nanomaterials make them ideal candidates for translation from the laboratory scale into the clinical arena for use in humans.

In recent years, plenty of researchers have reported that various gold nanomaterials could regulate the cell differentiation, maintain tissue stability and promote tissue regeneration in bone and cartilage. The present review focused on the biomedical applications and molecular mechanisms of gold nanomaterials in bone and cartilage TE. We described various kinds of gold nanomaterials and their cellular uptake process, followed by discussion on biomedical applications and molecular mechanisms of gold nanomaterials in bone and cartilage TE. The future work and perspective were also provided.

## Types of Gold Nanomaterials

The properties of gold nanomaterials depend sensitively on their size, shape, dimensionality and other properties. The types of gold nanomaterials can be classified according to these properties. Therefore, based on the dimensions of gold nanomaterials, they can be divided into several groups: zero-dimensional (0D), one-dimensional (1D), two-dimensional (2D), and three-dimensional (3D). Gold nanomaterials also can be sorted into various shapes such as gold nanospheres, nanocages, nanorods, nanobelts, nanosheets and so on (Dreaden et al., 2012; Dykman and Khlebtsov, 2012).

AuNPs, mostly referring to spherical gold nanomaterial, represent one type of 0D nanostructure materials possessing unique electrical, magnetic, optical and catalytic properties. AuNPs are widely used in medicine because of their excellent biocompatibility, low toxicity chemical stability and SPR detectability. They are easy to be surface-functionalized, and can be used more widely because of the ability to modify drugs, proteins, peptides and DNA (Mandal et al., 2011). Nanoparticles can easily penetrate the cell membrane and locate in the cytoplasm, thus affecting some cell signal pathways that induce differentiation (Li J. et al., 2017). It has been reported that when thiolated polyethylene glycol was coupled with AuNPs, AuNPs were able to be prevented from being attacked by the intravascular immune system, thus providing a biological basis for their use as drug carriers (Boisselier and Astruc, 2009). Recent studies have shown that AuNPs were closely related to bone and cartilage TE, especially in osteoblasts, osteoclasts, chondrocytes and bone marrow mesenchymal stem cells (BMSCs). Besides AuNPs in 0D nanomaterials, AuNCs and gold nanocages are also usually used in bone and cartilage TE due to their good stability, biocompatibility and two-photon absorption (Ramakrishna et al., 2008; Shiang et al., 2012).

In addition to 0D nanomaterials, AuNRs are an important 1D nanomaterials, that have plasma characteristics, unique optical properties, photoluminescence (Huang et al., 2009). 2D gold nanomaterials mainly include gold nanosheets, gold nanoplates and so on. For gold nanosheets, with thicknesses of single to few atomic layers, they have unique mechanical, electronic, and surface-related properties, which hold good application potential in the fields of photosensitive imaging, biological detection, catalysis and so on (Ye et al., 2019). However, compared to 0D nanomaterials especially AuNPs, the application of 1D and 2D gold nanomaterials in bone and cartilage TE deserves further study.

## Cellular Uptake Process of Gold Nanomaterials

It is known that gold nanomaterials need to perform their biological function by penetrating the cell membrane. Several uptake mechanisms were proposed and studied for gold nanomaterials. Cellular uptake ways of AuNPs are influenced by many factors, including shape, surface chemistry, functionalization and especially size (Chithrani et al., 2006). Li et al. (Li et al., 2016) suggested that AuNPs were taken in by human mesenchymal stem cells (hMSCs) in a size and shape-dependent manner. And AuNPs can enter into cells by various pathways such as phagocytosis, macropinocytosis, endocytosis and transcellular pathways (Zhao et al., 2011). In bone and cartilage TE, gold nanomaterials enter into cell mostly through endocytosis or transcellular pathway (Table 1). From the table, it could be inferred that endocytosis plays the most important role in bone and cartilage-related cellular uptake process of gold nanomaterials with or without functionalization. This effective uptake process keeps cell membrane intact and makes gold nanomaterials play a relatively stable function. Besides, gold nanomaterials are also able to play a role *via* binding to the membrane protein. In consideration of the toxicity and more secure applications, it is necessary to explore the cellular uptake mechanism of gold nanomaterials or the effect mechanism of extracellular gold nanomaterials in further related research.

**TABLE 1:** The main cellular uptake processes of gold nanomaterials in bone and cartilage tissue engineering.

Uptake pathways	Au nanomaterials used	TEM size (DLS size)	Cell or tissue used	Function	References
Direct diffusion	Chitosan-conjugated AuNPs	17 nm (40 nm)	hADMSCs	Osteogenic differentiation	Choi et al. (2015)
Endocytosis	AuNPs-loaded hydroxyapatite nanocomposites	4.7 ± 0.7 nm	hMSCs	Osteogenic differentiation	Liang et al. (2019)
Vesicles (endocytosis presumed)	AuNPs	(5, 13, 45 nm)	hPDLPs	Osteogenic differentiation	Zhang et al. (2017a)
Endocytosis	SPIO-Au core-shell NPs	17.3 ± 1.2 nm	Preosteoblast	Osteogenic differentiation	Yuan et al. (2017)
			MC3T3-E1 cells		
Endocytosis	AuNPs	20 nm (20 ± 2 nm)	Mice MSCs	Osteogenic differentiation	Yi et al. (2010)
Endocytosis	AuNPs, bisphosphonate-conjugated AuNPs	20–40 nm (20–49 nm)	BMMs	Inhibition of osteoclast differentiation	Lee et al. (2016)
Endocytosis/activating integrin pathway	AuNPs	20, 40 nm	Primary osteoblasts	Osteogenic differentiation	Zhang et al. (2014)
Endocytosis	Epigallocatechin gallate-functionalized AuNPs	30 nm (35.6 nm)	BMMs	Anti-osteoclastogenesis	Zhu et al. (2019)
Endocytosis	AuNPs, vitamin D-conjugated AuNPs	30–40 nm (36.5 ± 1.1, 60.8 ± 0.3 nm)	hADMSCs	Osteogenic differentiation	Nah et al. (2019)
Endocytosis (author presumed)	Gold nanosphere, nanostar, nanorod	40, 70, 110 nm	hMSCs	Osteogenesis	Li et al. (2016)
Vesicles (endocytosis presumed)	Human β-defensin 3-combined AuNPs	45 nm	hPDLCs	Osteogenic differentiation	Zhou et al. (2018)
Endocytosis	AuNPs	58.71 ± 22.33 nm	hPDLSC	Cell proliferation	Li et al. (2018a)
Endocytosis	AuNPs	13, 50 nm	Joints tissues	Antioxidants for collagen-induced arthritis	Kirdaite et al. (2019)
Endocytosis	Arginine-glycine-aspartate–modified AuNPs	39.4–41.9 nm (55.9–65.4 nm)	hMSCs	Chondrogenic differentiation	Li et al. (2017a)

BMMs, bone marrow-derived macrophages; DLS size, the size of nanomaterials in hydrodynamic form by dynamic light scattering; hADMSCs: human adipose-derived mesenchymal stem cells; hMSCs, human marrow mesenchymal stem cells; hPDLCs, human periodontal ligament cells; hPDLPCs, human periodontal ligament progenitor cells; hPDLSCs, human periodontal ligament stem cell; TEM size, the size of nanomaterials in dried form by transmission electron microscopy.

## Effect and Mechanism of Gold Nanomaterials in Bone and Cartilage Tissue Engineering

### Promotion and Regulation of the Differentiation

Bone marrow mesenchymal stem cells (BMSCs) are a heterogeneous population with high replication ability. They are pluripotent stem cells which can differentiate into osteoblasts and chondroblasts and then differentiate into bone or cartilage tissue. As mentioned above, BMSCs, one of three main factors, play a critical role in TE. The proliferation and differentiation of BMSCs can be considered as independent programmable processes, and controlling these processes in a predictable manner is crucial to regeneration of the desired tissue type. It has been reported that gold nanomaterials, mainly AuNPs, promoted osteogenic and chondrogenic differentiation through their effects on BMSCs (Yi et al., 2010; Sansanaphongpricha et al., 2020). The molecular mechanism mainly involved mitogen-activated protein kinase (MAPK), Wnt/β-catenin, and autophagy.

#### Regulation of Mitogen-Activated Protein Kinase Pathway

There are three parallel pathways of MAPK, including extracellular signal related kinases (ERK1/2), protein kinase 38 (p38), and c-Jun-N-terminal kinases (JNKs) pathways. ERK1/2 are generally referred to as growth factor-related kinases while p38 and JNK are often described as stress-activated protein kinases (SAPK2/p38 and SAPK1/JNK). Studies have indicated that MAPK pathway was involved in the proliferation and differentiation of osteoblasts (Greenblatt et al., 2013).

##### Extracellular Signal Related Kinases/Mitogen-Activated Protein Kinase Pathway

Among the three pathways, ERK/MAPK pathway is essential for cell growth and differentiation, which is also necessary for osteoblast adhesion, migration and integrin expression. In bone formation, ERK/MAPK pathway can transmit extracellular environmental information into the nucleus, which produces nuclear response to various of signals such as the stimulation of extracellular growth factors, extracellular matrix (ECM) or mechanical load (Ge et al., 2007).

**FIGURE 1:**
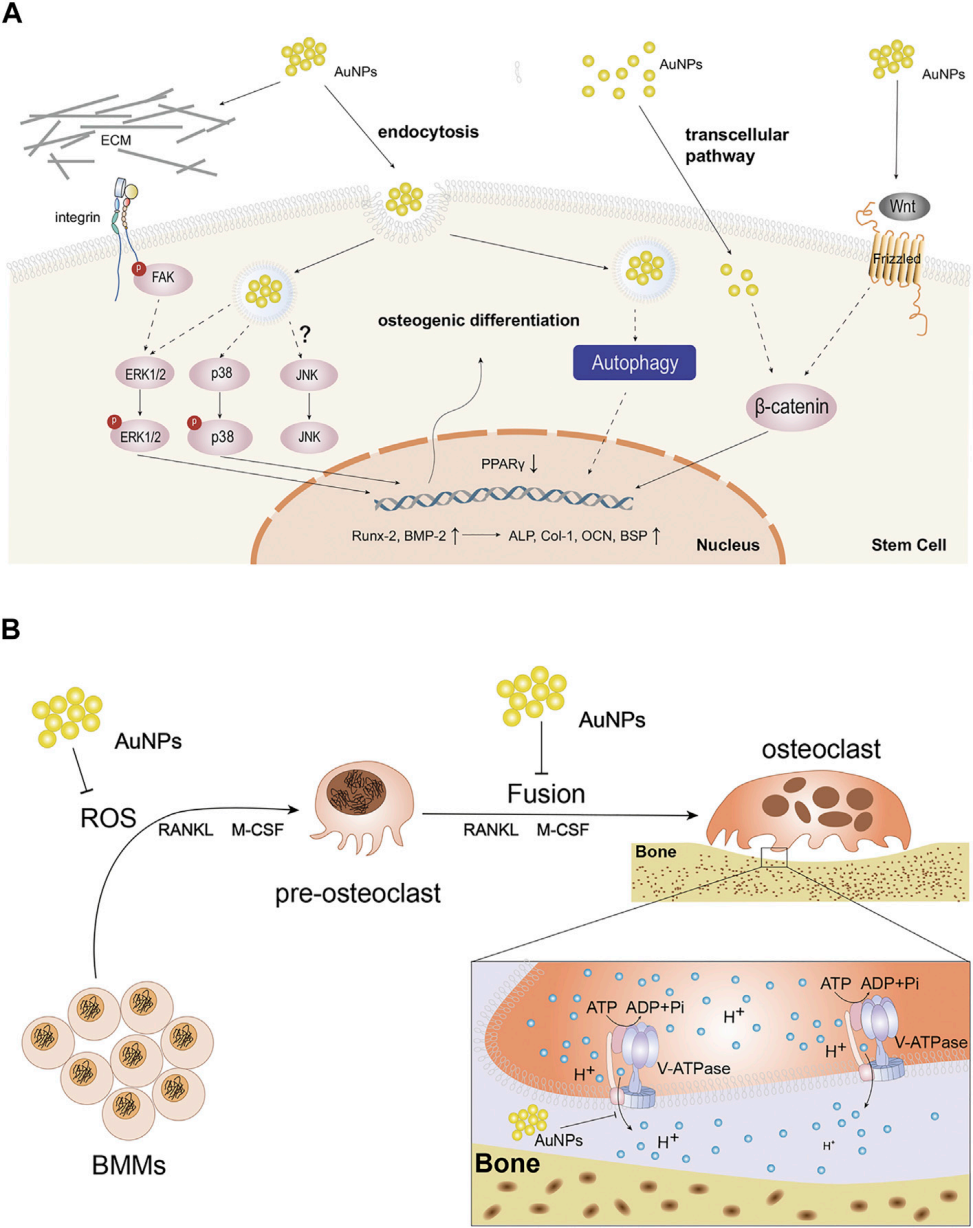
The function and molecular mechanism of AuNPs in cells. **(A)** Schematic diagram of the possible molecular pathways affected by the osteogenic effects of AuNPs. AuNPs activate integrin and Wnt signaling pathway extracellularly or enter cells through endocytosis and transcelluar pathway. AuNPs up-regulate Runx-2, BMP-2, ALP, Col-1, OCN, BSP and reduce PPARγ to enhance osteogenic differentiation via ERK/MAPK, P38/MAPK, Wnt/β-catenin signaling pathways and autophagy in osteogenesis-related cells. JNK/MAPK signaling pathway has been not reported so far. **(B)** Schematic representation of AuNPs disturbing the formation and function of osteoclasts. AuNPs inhibit ROS in BMMs, the fusion of pre-osteoclast cells or the function of V-ATPase in osteoclasts.

In the study of Zhang el al (Zhang et al., 2014), extracellular AuNPs can activate integrin of primary osteoblasts in answer to the chemical or physical changes in ECM and convert them into intracellular signals for mediating cell behavior. In detail, integrin then activated focal adhesion kinase (FAK) and ERK phosphorylation was enhanced in later, which finally activated ERK/MAPK pathway. Meanwhile, AuNPs also could enter cells via receptor-mediated endocytosis and directly or indirectly activate ERK/MAPK pathway (Figure 1A). Runt-related transcription factor-2 (Runx-2) was an vital mediator of MAPK reaction and previous studies indicated Runx-2 was a key transcription factor regulating the differentiation of BMSCs into osteoblasts due to the function directly regulating the expression of other osteoblastic specific genes including osteocalcin (OCN) and collagen type1 (Col-1), etc (Franceschi et al., 2003). Meanwhile, bone morphogenetic protein-2 (BMP-2) took most responsibility for osteoblasts differentiation (Mahmood et al., 2011) and could enhance the function of Runx-2. In a word, AuNPs activated ERK/MAPK pathway, up-regulated the expression of Runx2, BMP-2, OCN, Col-1 and increased the activity of the early marker for osteoblast differentiation, alkaline phosphatase (ALP) and the number of bone nodules, thus proving that AuNPs stimulate osteoblast proliferation and differentiation indeed through ERK/MAPK pathway.

##### Protein Kinase 38/Mitogen-Activated Protein Kinase Pathway

Among the three parallel MAPK pathways, p38 is usually called stress-activated protein kinase. Li et al.(Yi et al., 2010) demonstrated that AuNPs could interact with membrane of mesenchymal stem cells (MSCs) and enter into cells through receptor-mediated endocytosis, thus functionalizing as mechanical stimuli. In cells, AuNPs bound to the related-proteins and consequently activated the p38/MAPK signal pathway (Figure 1A). The study of Niu et al. (Niu et al., 2017) also discovered the same phenomenon in human periodontal ligament stem cells (hPDLSCs). Specifically, similar to ERK, AuNPs could up-regulate of osteogenic genes such as BMP-2, Runx-2, OCN, Col-1 and so on through the p38/MAPK signal pathway. In addition, AuNP could also down-regulate adipocyte major transcription factor peroxisome proliferator-activated receptor γ (PPARγ). In summary, AuNPs can produce mechanical stimulation and activate p38/MAPK signal pathway, thus up-regulating the expression of osteogenic genes and down-regulating the adipogenesis specific genes.

##### Jun-N-terminal kinases/Mitogen-Activated Protein Kinase Pathway

At present, there are no studies on AuNPs promoting osteogenic differentiation by activating JNK/MAPK pathway, but some studies have proved that up-regulating JNK expression and activating MAPK can promote osteogenic differentiation through insulin-like growth factor-1, mineral trioxide and other substances (Zhao et al., 2013; Wang et al., 2014). At the same time, it is not difficult to find that in most studies on JNK/MAPK pathway, JNK and ERK often play a role together (Kim et al., 2015). Whether these two pathways influence each other in the process of promoting osteogenic differentiation is worthy of further study. In addition, some studies have found that ERK, JNK and p38-MAPK were up-regulated under mechanical stimulation (Kyriakis and Avruch, 2001; Zhou et al., 2007). As mentioned above, AuNPs can activate p38/MAPK pathway through mechanical stress to promote osteogenesis, so whether AuNPs have similar effects on JNK pathway is worth exploring. In conclusion, AuNPs of a particular size and concentration could promote osteogenic differentiation in different kind of cells by MAPK pathways.

#### Regulation of Wnt/β-Catenin Pathway

The Wnt/β-catenin signaling pathway regulates the differentiation of progenitor cells into osteoblasts. Wnts are extracellular proteins that are crucial in multiple cellular functions and many studies have shown that the Wnt pathway was the powerful possible mechanism of AuNPs for promoting cell proliferation (Li et al., 2018a). Wnt signals are regulated by β-catenin that plays an important role in the signaling pathway. In the study of Seon Young choi (Choi et al., 2015), researchers used chitosan as a stabilizer for the reduction of AuNPs, and detected the increased expression of specific markers of osteogenic differentiation, such as OCN, bone sialoprotein (BSP) and ALP, in human adipose-derived mesenchymal stem cells (hADMSCs) co-cultured with chitosan-AuNPs, which confirmed the role of AuNPs in promoting osteogenic differentiation through Wnt/β-catenin pathway (Figure 1A). Subsequent study reported by Zhou et al. confirmed the above phenomenon in human periodontal ligament cells as well (Zhou et al., 2018).

Also, several researches suggested that mechanical stimulation enhanced osteogenesis and inhibited adipogenesis through activation of Wnt/β-catenin signaling (Chen et al., 2017). As mentioned earlier, AuNPs can interact with the cell membrane, enter the cell through endocytosis, and combine with proteins in the cytoplasm to produce mechanical stress. This unique mechanical stress activates Wnt/β-catenin pathway similar to the activation of MAPK pathway.

#### Regulation of Autophagy

Autophagy is a lysosome-based degradative pathway that responds to stress and maintains intracellular homeostasis, which is critical in various physiological and pathological process, including osteogenic differentiation. AuNPs have been shown to be a novel kind of autophagy modulators (Li et al., 2010). Therefore, the mechanism of AuNPs in osteogenic differentiation of MSCs involves autophagy (Figure 1A).

In the previous study, Zhang et al. (Zhang et al., 2017a) suggested that the osteogenic differentiation induced by AuNPs depends on the activation of autophagy. The early induction of autophagy is characterized by the accumulation of LC3-II binding to autophagosomes, the up-regulation of autophagy gene Beclin-1 and the decrease of selective autophagy target p62 (Mizushima et al., 2010). It has been proved that AuNPs can up-regulate the mRNA expression of LC3 and Beclin-1, and increase the activity and mineralization rate of ALP, which represented the degree of osteogenic differentiation. The osteogenic differentiation induced by AuNPs has a high similarity with natural osteogenic differentiation, which can play an effective role in bone TE while having little effect on other normal tissues, so they have greater potential in future application.

In addition, autophagy pathway also plays an important role in the differentiation and pathological changes of cartilage tissue. Autophagy has been shown to be related to cartilage formation, and studies have shown that inhibition of autophagy can lead to delayed cartilage development (Wang et al., 2015). However, there are few reports that gold nanomaterials regulate the process of cartilage differentiation by regulating the above pathways or cytokines, and the regulation of cartilage differentiation is mainly regulated by drugs, bioactive factors and so on.

### Protection for Bone and Cartilage Tissue

#### The Protection of Gold Nanoparticles in Bone Tissue

The protective effect of AuNPs in bone tissue is mainly through the inhibition of osteoclast. Osteoclasts, derived from monocyte/macrophage lineage cells, are the main functional cells for bone resorption and play the leading role in the balance between bone resorption and formation. The differentiation and proliferation of osteoclasts directly affect remodeling of bone tissue and hyperactive osteoclasts are the root cause for excessive bone resorption and subsequent osteoporosis. Studies have shown that AuNPs had the ability to inhibit osteoclast and were one of the most effective nanoparticles in the treatment of bone tissue diseases (Lee et al., 2016). Therefore, it is of great significance to clarify the regulatory mechanism of AuNPs on osteoclasts.

##### Inhibition of Receptor Activator of NF-κB Ligand-Induced Osteoclastogenesis

Receptor activator of NF-κB (nuclear factor-κB) ligand (RANKL) is a key factor motivating the differentiation and activation of osteoclasts. And RANKL is routinely used in the induction of osteoclast differentiation. Some studied have proved that AuNPs suppressed RANKL-induced osteoclastogenesis mainly by inhibiting reactive oxygen species (ROS) or the fusion of pre-osteoclast cells.

The binding of RANKL to its receptor RANK contributes to the recruitment of tumor necrosis factor receptor-related factor 6 (TRAF6) into the cytoplasmic domain of RANK. RANKL produces ROS that can act as a second messenger in bone marrow-derived macrophages (BMMs), which is involved in TRAF6, NADPH oxidase 1 (Nox1), Nox4 and Rac1. ROS then activate MAPK pathway that required for osteoclast differentiation (Atashi et al., 2015). Therefore, inhibition of ROS helps to inhibit osteoclast differentiation. Sul et al. (Sul et al., 2010) believed AuNPs, an antioxidant, inhibited RANLK-induced osteoclast formation in a dose-dependent manner at 1-2 ug/ml by attenuating ROS production and up-regulating the antioxidant enzyme glutathione peroxidase-1 (Gpx1) which prevented bone resorption (Figure 1B). Furthermore, it was believed that excessive ROS significantly restrained osteogenic signaling pathways while promoting adipogenic signaling pathways (Atashi et al., 2015). Therefore, AuNPs not only promotes osteogenesis, but also inhibits osteoclastogenesis through reducing ROS.

On the other hand, it was believed that nanoparticles could weaken RNAKL-induced osteoclastogenesis by suppressing pre-osteoclast fusion, including AuNPs (Zeng et al., 2019) (Figure 1B). Osteoclasts are multinuclear terminal cells formed via fusion of monocyte progenitors which is induced by RANKL and macrophage colony-stimulating factor (M-CSF) (Levaot et al., 2015). The fusion process involves three sequential steps. Firstly, BMMs are induced to form pre-osteoclasts possessing the ability to fusion induced by RANKL and M-CSF. Then, these cells migrate, aggregate and adhere to each other with their plasma membranes. Finally, the continuous fusion of pre-osteoclasts forms multinucleated cells, namely osteoclasts. In osteoclastogenesis, cell fusion is a necessary and rate-limiting step in osteoclast development. Li et al. (Zeng et al., 2019) demonstrated that AuNPs suppressed pre-osteoclast migration in a dose-dependent manner and prevented pre-osteoclast fusion by down-regulating expression of fusogenic genes, such as Cx43. In summary, AuNPs can suppress RANKL-induced osteoclastogenesis and they are expected to be a potential target for the treatment of excessive bone resorption.

##### Regulation of Acidic Microenvironment

AuNPs not only inhibit the generation of osteoclasts, but also disturb the function of osteoclasts. Osteoclasts degrade bone via lysosomal proteases activated in acidic microenvironment which are controlled by vacuolar-type H^+^-ATPase (V-ATPase) (Sun-Wada et al., 2003). In osteoclasts, V-ATPase can transfer protons across membranes into extracellular microenvironment and ultimately create an acidic condition. It has been demonstrated that V-ATPase mutations can contribute to paralysis of osteoclasts (Bhargava et al., 2012). In previous studies, AuNPs could prevent V-ATPase binding on the endosome membrane and inhibit the function of V-ATPase, thus alkalifying microenvironment and deactivating osteoclasts (Bai et al., 2018). Further research also suggested that AuNPs can obstruct acidification of osteoclast absorption microenvironment through directly disturbing the partial domain of V-ATPase (Bai et al., 2020) (Figure 1B). However, whether the interference of AuNPs on the activity of V-ATP enzyme will cause abnormality of other metabolic processes in osteoclasts and bone tissue, and whether other enzymes similar to V-ATPase structure or function will be inhibited by AuNPs remain to be further explored.

#### The Protection of Gold Nanoclusters in Bone Tissue

Gold nanoclusters (AuNCs) as emerging fluorescent nanomaterials are smaller than nanoparticles and have better biocompatibility in bone and cartilage. In the study of Kuo Li (Li K. et al., 2020), they constructed AuNCs protected by ultra-small lysozyme (Lys) and found that Lys-AuNCs could not only promote osteogenic differentiation, but also inhibit the formation of osteoclasts. More importantly, the study showed that lysozyme itself had no significant effect on improving the viability of MC3T3E1, important cells for bone tissue regeneration, and the main reason for promoting proliferation rate was the existence of AuNCs. However, except AuNPs and AuNCs, there are few in-depth studies for other types of gold nanomaterials in bone TE and in consideration of their properties, further subsequent studies are needed.

#### The Protection for Cartilage Tissue

Cartilage tissues with lubrication and cushioning effects are mainly responsible for large mechanical loads (Gilbert and Blain, 2018). As thus, cartilage tissues are easy to be damaged. However, the repair of impaired cartilage is always challenging. The protective function of gold nanomaterials in cartilage tissue has great application prospect, particularly in articular cartilage tissue, including rheumatic arthritis (RA) and OA.

In many studies, it has been demonstrated that AuNPs can inhibit angiogenic activities, suppress inflammation or serve as antioxidant to protect cartilage tissue in arthritis. In the study of Tsai et al. (Tsai et al., 2007), AuNPs have been shown to alleviate collagen-induced arthritis which imitated RA in humans for the first time. They could bound to vascular endothelial growth factor (VEGF), an angiogenic factor, and inhibit endothelial cell proliferation and migration. Another study also indicated that AuNPs in the form of complexes bound to VEGF to treat RA (Lee et al., 2014). Furthermore, AuNPs could down-regulate pro-inflammatory responses and consequently inhibit inflammation both in OA and RA (Gomes et al., 2016; Gul et al., 2018). And the protection of AuNPs for ECM of which the degradation is related to OA and RA have been proved. AuNPs can also quench ROS and prevent the destruction of synovitis in RA (Kirdaite et al., 2019). At the same time, AuNPs as carriers can play a good role in cartilage TE, which will be discussed in the following section.

Similar to AuNPs, gold nanocages are a kind of porous nanogold materials with good biocompatibility to easily use in combination with other materials. And they could reduce synovial hyperplasia and protect cartilage (Wang et al., 2020). As for AuNRs, it is also easy to modify the surface of their surface. And with the help of other drugs or protein, AuNRs have been shown to promote chondrogenesis (Sansanaphongpricha et al., 2020). As can be seen from the examples above, gold nanomaterials are not only useful as therapeutic agents for cartilage disease but also have enormous potential in cartilage TE.

### Carriers in Bone and Cartilage Tissue Engineering

Gold nanomaterials have been widely used in biomedical fields including delivery carriers, because of their adjustable size, optical properties, as well as their biocompatibility (Lopes et al., 2019). Their deliverable application can range from small drug molecules to large biomolecules, such as proteins, DNA or RNA. Among them, AuNPs have become a promising platform for a variety of biomedical applications due to the characteristics of easy synthesis, easy functionalization, stable properties, non-toxic and so on (Boisselier and Astruc, 2009). Similar to AuNPs, AuNRs are also easy for surface modifications and ready to conjugate with functionalized polymers, antibodies and peptides (Huang et al., 2009; Bartczak et al., 2011). Therefore, in bone and cartilage TE, gold nanomaterials could serve as carriers in small molecules and biomacromolecules reputably and need pay more attention to clinic application study.

#### Small Molecules Delivery

Prior to acting as drug carriers, AuNPs are usually functionalized, which could enhance the stability of AuNPs, increase the circulation time, reduce their side effect, strengthen their interactional ability, improve their biocompatibility and reinforce the regional orientation of drug action. At present, the commonly used functionalization techniques include surface modification via mercaptan, polyethylene glycol (PEG), amino acid (Dykman and Khlebtsov, 2012) and combination of AuNPs with other biocompatible materials (Li H. et al., 2020). With above techniques, it is generally believed that AuNPs have good performance in drug delivery.

Heo et al. (Heo et al., 2014) combined curcumin with AuNPs to prepare cyclodextrin β-cyclodextrin coupled AuNPs, which could significantly restrain the formation of tartrate-resistant acid phosphatase (TRAP)-positive multinucleated cells safely in BMMs. Nah et al. (Nah et al., 2019) used AuNPs to carry vitamin D. The developed AuNPs-vitamin D complex combined well with the mercaptan group between AuNPs and vitamin D. Through the detection of related data, it was confirmed that the complex enhanced osteogenic differentiation. Some researchers also have shown that AuNPs-loaded hydroxyapatite can be internalized into hMSCs and enhance the osteogenic differentiation of hMSCs (Liang et al., 2019). In the above examples, it can be found that AuNPs can carry many types of drugs, which can protect the biological activity of the delivered drugs while improving the bone tissue targeting of the drugs. At the same time, they can play a synergistic effect with the delivered drugs to improve the efficiency of bone TE.

In cartilage tissue, AuNPs can also play the role of drug delivery. Intra-articular injection provides a highly effective and low systemic side effect for the treatment of joint diseases, but it cannot keep the efficacy caused by reduced drug concentration. The application of AuNPs can solve this problem to some extent. Its advantage is that AuNPs can be designed in different sizes and combine with drugs, thus slowing down the clearance of drugs, and when combined with AuNPs, drugs can penetrate ECM or cell barrier (Dwivedi et al., 2015).

The reason that the nano-drug loading system can attract so much attention is mainly due to its unique performance and characteristics in the delivery of drugs for the treatment of diseases. By the way, the use of AuNPs as drug carriers can protect drugs from attacked by the human immune system, and have the characteristics of large drug loading. But at the same time, they are restricted by their limited biodegradability.

#### Biomacromolecules Delivery

In addition to small molecular drugs, AuNPs can also deliver large biomolecules. In practical work, researchers used a variety of ways to functionalize AuNPs, and utilized functionalized AuNPs to carry the required biological macromolecules. Their adjustable size and function make them a useful scaffold for effective recognition and transmission of biomolecules. At present, it has been proved that AuNPs were successful in the delivery of peptides, proteins or nucleic acids, such as DNA or RNA (Graczyk et al., 2020).

In the field of bone and cartilage TE, it has been reported that AuNPs can deliver siRNA, miRNA and other biomacromolecules with the assistance of various modification (Pan et al., 2016; Wu et al., 2020). AuNPs as effective biomacromolecules delivery carriers provide adequate protection to prevent them from being degraded by enzymes. Certainly, the function of above molecules was enhanced by the carriers. Similarly, AuNRs could serve as delivery carries of BMP-2 and siRNA and ultimately promote osteogenesis and chondrogenesis (Tsai et al., 2007; Zhao et al., 2015; Sansanaphongpricha et al., 2020). In summary, there is massive clinical potential for gold nanomaterials as carrier in bone and cartilage TE.

## Conclusions and Future Perspective

The effective combination of nano-bioengineering and regenerative medicine has become the focus of international research. After establishing interdisciplinary nanotechnology fields, it may be believed that nanomaterials will combine with chemically and clinically applicable fields in the next generation of chemical and medical platforms. Gold nanomaterials, as a new type of medical materials, play a direct role as drug or indirect role as drug carriers. They not only promoted the progress of alternative medical methods, but also provide new ideas and new goals for the clinical treatment for bone and cartilage diseases represented by osteoporosis, bone defect and arthritis. In this review, the majority of biomedical mechanism and applications demonstrated that gold nanomaterials, especially AuNPs, had a protective effect on bone and cartilage tissue and could be further modified to promote delivery efficiency and loading with other drugs or biomacromolecules. However, it is worth noting that gold nanomaterials need further examination in clinical studies, which may obviously provide help for reducing the medical pressure on bone and cartilage diseases, increasingly with a rapidly aging population.

In spite of the potential therapeutic effectiveness of gold nanomaterials, several limitations and deficiencies of their applications remain. In bone and cartilage TE, the application of gold nanomaterials is mainly limited to 0D AuNPs and 1D AuNRs. However, while 2D gold nanosheets and other hierarchical gold nanomaterials with different and unique physical and chemical properties are well used in different directions, such as tumor treatment, examination, imaging and so on, there are few reports on the application of these materials in bone and cartilage TE. It is thus highly desirable to explore their utilization due to the broad research prospects. In addition, whether naked gold nanomaterials can have a therapeutic effect on chondrocytes and cartilage tissue needs to be further clarified with potential mechanism.

## References

[B1] Agarwal R. García A. J. (2015). Biomaterial Strategies for Engineering Implants for Enhanced Osseointegration and Bone Repair. Adv. Drug Deliv. Rev. 94, 53–62. 10.1016/j.addr.2015.03.013 25861724 PMC4598264

[B2] Atashi F. Modarressi A. Pepper M. S. (2015). The Role of Reactive Oxygen Species in Mesenchymal Stem Cell Adipogenic and Osteogenic Differentiation: a Review. Stem Cell Develop. 24 (10), 1150–1163. 10.1089/scd.2014.0484 PMC4424969 25603196

[B3] Bai X. Gao Y. Zhang M. Chang Y.-n. Chen K. Li J. (2020). Carboxylated Gold Nanoparticles Inhibit Bone Erosion by Disturbing the Acidification of an Osteoclast Absorption Microenvironment. Nanoscale 12 (6), 3871–3878. 10.1039/c9nr09698a 31996882

[B4] Bai X. Zhang J. Chang Y.-N. Gu W. Lei R. Qin Y. (2018). Nanoparticles with High-Surface Negative-Charge Density Disturb the Metabolism of Low-Density Lipoprotein in Cells. Int J. Mol. Sci 19 (9), 2790. 10.3390/ijms19092790 PMC6164102 30227604

[B5] Bartczak D. Kanaras A. G. Colloids (2011). Preparation of Peptide-Functionalized Gold Nanoparticles Using One Pot EDC/Sulfo-NHS Coupling. Langmuir 27 (16), 10119–10123. 10.1021/la2022177 21728291

[B6] Bhargava A. Voronov I. Wang Y. Glogauer M. Kartner N. Manolson M. F. (2012). Osteopetrosis Mutation R444L Causes Endoplasmic Reticulum Retention and Misprocessing of Vacuolar H+-ATPase A3 Subunit. J. Biol. Chem. 287 (32), 26829–26839. 10.1074/jbc.M112.345702 22685294 PMC3411020

[B7] Boisselier E. Astruc D. (2009). Gold Nanoparticles in Nanomedicine: Preparations, Imaging, Diagnostics, Therapies and Toxicity. Chem. Soc. Rev. 38 (6), 1759–1782. 10.1039/b806051g 19587967

[B8] Chen X. Guo J. Yuan Y. Sun Z. Chen B. Tong X. (2017). Cyclic Compression Stimulates Osteoblast Differentiation via Activation of the Wnt/β-Catenin Signaling Pathway. Mol. Med. Rep. 15 (5), 2890–2896. 10.3892/mmr.2017.6327 28447744

[B9] Chithrani B. D. Ghazani A. A. Chan W. C. W. (2006). Determining the Size and Shape Dependence of Gold Nanoparticle Uptake into Mammalian Cells. Nano Lett. 6 (4), 662–668. 10.1021/nl052396o 16608261

[B10] Choi S. Y. Song M. S. Ryu P. D. Lam A. T. Joo S. W. Lee S. Y. (2015). Gold Nanoparticles Promote Osteogenic Differentiation in Human Adipose-Derived Mesenchymal Stem Cells through the Wnt/β-Catenin Signaling Pathway. Int. J. Nanomedicine 10, 4383–4392. 10.2147/IJN.S78775 26185441 PMC4500612

[B11] Dreaden E. C. Alkilany A. M. Huang X. Murphy C. J. El-Sayed M. A. (2012). The golden Age: Gold Nanoparticles for Biomedicine. Chem. Soc. Rev. 41 (7), 2740–2779. 10.1039/c1cs15237h 22109657 PMC5876014

[B12] Du X. Zhang X. Jiang C. Zhang W. Yang L. (2018). The Trace Detection of Nitrite Ions Using Neutral Red Functionalized SH-β-Cyclodextrin @Au Nanoparticles. Sensors 18 (3), 681. 10.3390/s18030681 PMC5877208 29495331

[B13] Dwivedi P. Nayak V. Kowshik M. (2015). Role of Gold Nanoparticles as Drug Delivery Vehicles for Chondroitin Sulfate in the Treatment of Osteoarthritis. Biotechnol. Prog. 31 (5), 1416–1422. 10.1002/btpr.2147 26193993

[B14] Dykman L. Khlebtsov N. (2012). Gold Nanoparticles in Biomedical Applications: Recent Advances and Perspectives. Chem. Soc. Rev. 41 (6), 2256–2282. 10.1039/c1cs15166e 22130549

[B15] Franceschi R. Xiao G. Jiang D. Gopalakrishnan R. Yang S. Reith E. (2003). Multiple Signaling Pathways Converge on the Cbfa1/Runx2 Transcription Factor to Regulate Osteoblast Differentiation. Connect. Tissue Res. 44 (1), 109–116. 10.1080/713713603 PMC3564252 12952183

[B16] Gao H. Xiang W. Ma X. Ma L. Huang Y. Ni H. (2015). Sol-gel Synthesis and Third-Order Optical Nonlinearity of Au Nanoparticles Doped Monolithic Glass. Gold Bull. 48 (3-4), 153–159. 10.1007/s13404-015-0173-1

[B17] Ge C. Xiao G. Jiang D. Franceschi R. T. (2007). Critical Role of the Extracellular Signal-Regulated Kinase-MAPK Pathway in Osteoblast Differentiation and Skeletal Development. J. Cel Biol 176 (5), 709–718. 10.1083/jcb.200610046 PMC2064027 17325210

[B18] Gilbert S. J. Blain E. J. (2018). Cartilage Mechanobiology: How Chondrocytes Respond to Mechanical Load, London: Academic Press, 99–126. 10.1016/b978-0-12-812952-4.00004-0

[B19] Gomes A. Saha P. Bhowmik T. Dasgupta A. Dasgupta S. (2016). Protection against Osteoarthritis in Experimental Animals by Nanogold Conjugated Snake Venom Protein Toxin Gold Nanoparticle-Naja Kaouthia Cytotoxin 1. Indian J. Med. Res. 144 (6), 910–917. 10.4103/ijmr.IJMR_1078_14 28474628 PMC5433284

[B20] Graczyk A. Pawlowska R. Jedrzejczyk D. Chworos A. (2020). Gold Nanoparticles in Conjunction with Nucleic Acids as a Modern Molecular System for Cellular Delivery. Molecules 25 (1), 204. 10.3390/molecules25010204 PMC6982881 31947834

[B21] Greenblatt M. B. Shim J.-H. Glimcher L. H. (2013). Mitogen-activated Protein Kinase Pathways in Osteoblasts. Annu. Rev. Cel Dev. Biol. 29, 63–79. 10.1146/annurev-cellbio-101512-122347 23725048

[B22] Gul A. Kunwar B. Mazhar M. Faizi S. Ahmed D. Shah M. R. (2018). Rutin and Rutin-Conjugated Gold Nanoparticles Ameliorate Collagen-Induced Arthritis in Rats through Inhibition of NF-Κb and iNOS Activation. Int. Immunopharmacology 59, 310–317. 10.1016/j.intimp.2018.04.017 29679855

[B23] He B. Wang Y. Zhai Q. Qiu P. Dong G. Liu X. (2020). From Polymeric Carbon Nitride to Carbon Materials: Extended Application to Electrochemical Energy Conversion and Storage. Nanoscale 12 (16), 8636–8646. 10.1039/d0nr01612h 32296803

[B24] Heo D. N. Ko W.-K. Moon H.-J. Kim H.-J. Lee S. J. Lee J. B. (2014). Inhibition of Osteoclast Differentiation by Gold Nanoparticles Functionalized with Cyclodextrin Curcumin Complexes. ACS Nano 8 (12), 12049–12062. 10.1021/nn504329u 25420230

[B25] Hu X. Huang J. Zhao F. Yi P. He B. Wang Y. (2020). Photothermal Effect of Carbon Quantum Dots Enhanced Photoelectrochemical Water Splitting of Hematite Photoanodes. J. Mater. Chem. A. 8 (30), 14915–14920. 10.1039/d0ta04144k

[B26] Huang J. Chen T. Zhao M. Yi P. Zhao F. He B. (2021). Surface Oxygen Vacancies of TiO2 Nanorods by Electron Beam Irradiation for Efficient Photoelectrochemical Water Splitting. CrystEngComm 23 (16), 2952–2960. 10.1039/d1ce00205h

[B27] Huang X. Neretina S. El-Sayed M. A. J. A. M. (2009). Gold Nanorods: From Synthesis and Properties to Biological and Biomedical Applications. Adv. Mater. 21, 4880–5491. 10.1002/adma.200802789 25378252

[B28] Huang Y. Lin D. Li M. Yin D. Wang S. Wang J. (2019). Ag@Au Core-Shell Porous Nanocages with Outstanding SERS Activity for Highly Sensitive SERS Immunoassay. Sensors 19 (7), 1554. 10.3390/s19071554 PMC6480069 30935114

[B29] Huang Y. Xiang W. Lin S. Cao R. Zhang Y. Zhong J. (2017). The Synthesis of Bimetallic Gold Plus Nickel Nanoparticles Dispersed in a Glass Host and Behavior-Enhanced Optical Nonlinearities. J. Non-Crystalline Sol. 459, 142–149. 10.1016/j.jnoncrysol.2017.01.007

[B30] Jiang B. Xu L. Chen W. Zou C. Yang Y. Fu Y. (2017). Ag+-assisted Heterogeneous Growth of Concave Pd@Au Nanocubes for Surface Enhanced Raman Scattering (SERS). Nano Res. 10 (10), 3509–3521. 10.1007/s12274-017-1562-y

[B31] Jiang K. Wang J. Wu F. Xue Q. Yao Q. Zhang J. (2020). Dopant‐Free Organic Hole‐Transporting Material for Efficient and Stable Inverted All‐Inorganic and Hybrid Perovskite Solar Cells. Adv. Mater. 32 (16), 1908011. 10.1002/adma.201908011 32115824

[B32] Jiang X.-M. Wang L.-M. Wang J. Chen C.-Y. (2012). Gold Nanomaterials: Preparation, Chemical Modification, Biomedical Applications and Potential Risk Assessment. Appl. Biochem. Biotechnol. 166 (6), 1533–1551. 10.1007/s12010-012-9548-4 22278050

[B33] Keeney M. Lai J. H. Yang F. (2011). Recent Progress in Cartilage Tissue Engineering. Curr. Opin. Biotechnol. 22 (5), 734–740. 10.1016/j.copbio.2011.04.003 21531126

[B34] Kim B. S. Kang H.-J. Park J.-Y. Lee J. (2015). Fucoidan Promotes Osteoblast Differentiation via JNK- and ERK-dependent BMP2-Smad 1/5/8 Signaling in Human Mesenchymal Stem Cells. Exp. Mol. Med. 47, e128. 10.1038/emm.2014.95 25572360 PMC4314586

[B35] Kirdaite G. Leonaviciene L. Bradunaite R. Vasiliauskas A. Rudys R. Ramanaviciene A. (2019). Antioxidant Effects of Gold Nanoparticles on Early Stage of Collagen-Induced Arthritis in Rats. Res. Vet. Sci. 124, 32–37. 10.1016/j.rvsc.2019.02.002 30807910

[B36] Kyriakis J. M. Avruch J. (2001). Mammalian Mitogen-Activated Protein Kinase Signal Transduction Pathways Activated by Stress and Inflammation. Physiol. Rev. 81 (2), 807–869. 10.1152/physrev.2001.81.2.807 11274345

[B37] Lee D. Heo D. N. Kim H.-J. Ko W.-K. Lee S. J. Heo M. (2016). Inhibition of Osteoclast Differentiation and Bone Resorption by Bisphosphonate-Conjugated Gold Nanoparticles. Sci. Rep. 6, 27336. 10.1038/srep27336 27251863 PMC4890291

[B38] Lee H. Lee M.-Y. Bhang S. H. Kim B.-S. Kim Y. S. Ju J. H. (2014). Hyaluronate-gold Nanoparticle/tocilizumab Complex for the Treatment of Rheumatoid Arthritis. ACS Nano 8 (5), 4790–4798. 10.1021/nn500685h 24730974

[B39] Levaot N. Ottolenghi A. Mann M. Guterman-Ram G. Kam Z. Geiger B. (2015). Osteoclast Fusion Is Initiated by a Small Subset of RANKL-Stimulated Monocyte Progenitors, Which Can Fuse to RANKL-Unstimulated Progenitors. Bone 79, 21–28. 10.1016/j.bone.2015.05.021 26008608

[B40] Li C. Li Z. Zhang Y. Fathy A. H. Zhou M. (2018a). The Role of the Wnt/β-Catenin Signaling Pathway in the Proliferation of Gold Nanoparticle-Treated Human Periodontal Ligament Stem Cells. Stem Cel Res Ther 9 (1), 214. 10.1186/s13287-018-0954-6 PMC6085621 30092818

[B41] Li C. Zhang Y. Li Z. Mei E. Lin J. Li F. (2018b). Light-Responsive Biodegradable Nanorattles for Cancer Theranostics. Adv. Mater. 30 (8), 1706150. 10.1002/adma.201706150 29271515

[B42] Li H. Pan S. Xia P. Chang Y. Fu C. Kong W. (2020a). Advances in the Application of Gold Nanoparticles in Bone Tissue Engineering. J. Biol. Eng. 14 (1). 10.1186/s13036-020-00236-3 PMC7201659 32391080

[B43] Li J. J. Hartono D. Ong C.-N. Bay B.-H. Yung L.-Y. L. (2010). Autophagy and Oxidative Stress Associated with Gold Nanoparticles. Biomaterials 31 (23), 5996–6003. 10.1016/j.biomaterials.2010.04.014 20466420

[B44] Li J. Li J. E. J. Zhang J. Wang X. Kawazoe N. Chen G. (2016). Gold Nanoparticle Size and Shape Influence on Osteogenesis of Mesenchymal Stem Cells. Nanoscale 8 (15), 7992–8007. 10.1039/c5nr08808a 27010117

[B45] Li J. Li X. Zhang J. Kawazoe N. Chen G. (2017a). Induction of Chondrogenic Differentiation of Human Mesenchymal Stem Cells by Biomimetic Gold Nanoparticles with Tunable RGD Density. Adv. Healthc. Mater. 6 (14), 1700317. 10.1002/adhm.201700317 28489328

[B46] Li K. Zhuang P. Tao B. Li D. Xing X. Mei X. (2020b). Ultra-Small Lysozyme-Protected Gold Nanoclusters as Nanomedicines Inducing Osteogenic Differentiation. Int. J. Nanomedicine 15, 4705–4716. 10.2147/IJN.S241163 32636626 PMC7335297

[B47] Li N.-M. Li K.-M. Wang S. Yang K.-Q. Zhang L.-J. Chen Q. (2015). Gold Embedded Maghemite Hybrid Nanowires and Their Gas Sensing Properties. ACS Appl. Mater. Inter. 7 (19), 10534–10540. 10.1021/acsami.5b02087 25938724

[B48] Li Q. Wang F. Bai Y. Xu L. Yang Y. Yan L. (2017b). Decahedral-shaped Au Nanoparticles as Plasmonic Centers for High Performance Polymer Solar Cells. Org. Electron. 43, 33–40. 10.1016/j.orgel.2017.01.010

[B49] Li X. Yang Y. Zhou G. Han S. Wang W. Zhang L. (2013). The Unusual Effect of AgNO3 on the Growth of Au Nanostructures and Their Catalytic Performance. Nanoscale 5 (11), 4976–4985. 10.1039/c3nr00603d 23636467

[B50] Liang H. Xu X. Feng X. Ma L. Deng X. Wu S. (2019). Gold Nanoparticles-Loaded Hydroxyapatite Composites Guide Osteogenic Differentiation of Human Mesenchymal Stem Cells through Wnt/β-Catenin Signaling Pathway. Int. J. Nanomedicine 14, 6151–6163. 10.2147/IJN.S213889 31447557 PMC6683960

[B51] Lopes T. S. Alves G. G. Pereira M. R. Granjeiro J. M. Leite P. E. C. (2019). Advances and Potential Application of Gold Nanoparticles in Nanomedicine. J. Cel Biochem 120 (10), 16370–16378. 10.1002/jcb.29044 31127662

[B52] Ma Y. Xu L. Chen W. Zou C. Yang Y. Zhang L. (2015). Evolution from Small Sized Au Nanoparticles to Hollow Pt/Au Nanostructures with Pt Nanorods and a Mechanistic Study. RSC Adv. 5 (126), 103797–103802. 10.1039/c5ra21807a

[B53] Mahmood A. Napoli C. Aldahmash A. (2011). In VitroDifferentiation and Maturation of Human Embryonic Stem Cell into Multipotent Cells. Stem Cell Int. 2011, 735420. 10.4061/2011/735420 PMC3154539 21845195

[B54] Mandal S. Bakeine G. J. Krol S. Ferrari C. Clerici A. M. Zonta C. (2011). Design, Development and Characterization of Multi-Functionalized Gold Nanoparticles for Biodetection and Targeted boron Delivery in BNCT Applications. Appl. Radiat. Isot. 69 (12), 1692–1697. 10.1016/j.apradiso.2011.05.002 21641810

[B55] Mizushima N. Yoshimori T. Levine B. (2010). Methods in Mammalian Autophagy Research. Cell 140 (3), 313–326. 10.1016/j.cell.2010.01.028 20144757 PMC2852113

[B56] Nah H. Lee D. Heo M. Lee J. S. Lee S. J. Heo D. N. (2019). Vitamin D-Conjugated Gold Nanoparticles as Functional Carriers to Enhancing Osteogenic Differentiation. Sci. Technol. Adv. Mater. 20 (1), 826–836. 10.1080/14686996.2019.1644193 31489055 PMC6713151

[B57] Niu C. Yuan K. Ma R. Gao L. Jiang W. Hu X. (2017). Gold Nanoparticles Promote Osteogenic Differentiation of Human Periodontal Ligament Stem Cells via the P38 MAPK Signaling Pathway. Mol. Med. Rep. 16 (4), 4879–4886. 10.3892/mmr.2017.7170 28791361

[B58] Pan S. Li J. Wen Z. Lu R. Zhang Q. Jin H. (2021). Halide Perovskite Materials for Photo(Electro)Chemical Applications: Dimensionality, Heterojunction, and Performance. Adv. Energ. Mater., 2004002. 10.1002/aenm.202004002

[B59] Pan T. Song W. Gao H. Li T. Cao X. Zhong S. (2016). miR-29b-Loaded Gold Nanoparticles Targeting to the Endoplasmic Reticulum for Synergistic Promotion of Osteogenic Differentiation. ACS Appl. Mater. Inter. 8 (30), 19217–19227. 10.1021/acsami.6b02969 27399270

[B60] Ramakrishna G. Varnavski O. Kim J. Lee D. Goodson T. (2008). Quantum-sized Gold Clusters as Efficient Two-Photon Absorbers. J. Am. Chem. Soc. 130 (15), 5032–5033. 10.1021/ja800341v 18357982

[B61] Sansanaphongpricha K. Sonthithai P. Kaewkong P. Thavornyutikarn B. Bamrungsap S. Kosorn W. (2020). Hyaluronic Acid-Coated Gold Nanorods Enhancing BMP-2 Peptide Delivery for Chondrogenesis. Nanotechnology 31 (43), 435101. 10.1088/1361-6528/aba46d 32647102

[B62] Shiang Y.-C. Huang C.-C. Chen W.-Y. Chen P.-C. Chang H.-T. (2012). Fluorescent Gold and Silver Nanoclusters for the Analysis of Biopolymers and Cell Imaging. J. Mater. Chem. 22 (26), 12972–12982. 10.1039/c2jm30563a

[B63] Sul O.-J. Kim J.-C. Kyung T.-W. Kim H.-J. Kim Y.-Y. Kim S.-H. (2010). Gold Nanoparticles Inhibited the Receptor Activator of Nuclear Factor-Κb Ligand (RANKL)-Induced Osteoclast Formation by Acting as an Antioxidant. Biosci. Biotechnol. Biochem. 74 (11), 2209–2213. 10.1271/bbb.100375 21071867

[B64] Sun-Wada G.-H. Wada Y. Futai M. (2003). Vacuolar H+ Pumping ATPases in Luminal Acidic Organelles and Extracellular Compartments: Common Rotational Mechanism and Diverse Physiological Roles. J. Bioenerg. biomembranes 35 (4), 347–358. 10.1023/a:1025780932403 14635780

[B65] Tsai C.-Y. Shiau A.-L. Chen S.-Y. Chen Y.-H. Cheng P.-C. Chang M.-Y. (2007). Amelioration of Collagen-Induced Arthritis in Rats by Nanogold. Arthritis Rheum. 56 (2), 544–554. 10.1002/art.22401 17265489

[B66] Wang B. Iocozzia J. Zhang M. Ye M. Yan S. Jin H. (2019). The Charge Carrier Dynamics, Efficiency and Stability of Two-Dimensional Material-Based Perovskite Solar Cells. Chem. Soc. Rev. 48 (18), 4854–4891. 10.1039/c9cs00254e 31389932

[B67] Wang X. Qi H. Wang Q. Zhu Y. Wang X. Jin M. (2015). FGFR3/fibroblast Growth Factor Receptor 3 Inhibits Autophagy through Decreasing the ATG12–ATG5 Conjugate, Leading to the Delay of Cartilage Development in Achondroplasia. Autophagy 11 (11), 1998–2013. 10.1080/15548627.2015.1091551 26491898 PMC4824585

[B68] Wang Y. Li J. Song W. Yu J. (2014). Mineral Trioxide Aggregate Upregulates Odonto/osteogenic Capacity of Bone Marrow Stromal Cells from Craniofacial bonesviaJNK and ERK MAPK Signalling Pathways. Cell Prolif. 47 (3), 241–248. 10.1111/cpr.12099 24635197 PMC6496412

[B69] Wang Z. Yang J. Yang Y. Pu X. Zhao J. Zhang N. (2020). Targeted and Combined TPCA-1-Gold Nanocage Therapy for *In Vivo* Treatment of Inflammatory Arthritis. AAPS PharmSciTech 21 (8), 298. 10.1208/s12249-020-01856-0 33140225

[B70] Wu Q. Wang K. Wang X. Liang G. Li J. (2020). Delivering siRNA to Control Osteogenic Differentiation and Real-Time Detection of Cell Differentiation in Human Mesenchymal Stem Cells Using Multifunctional Gold Nanoparticles. J. Mater. Chem. B 8 (15), 3016–3027. 10.1039/c9tb02899d 32207489

[B71] Xu L. Wang K. Jiang B. Chen W. Liu F. Hao H. (2016). Competitive Effect in the Growth of Pd-Au-Pd Segmental Nanorods. Chem. Mater. 28 (20), 7394–7403. 10.1021/acs.chemmater.6b02871

[B72] Ye S. Brown A. P. Stammers A. C. Thomson N. H. Wen J. Roach L. (2019). Sub‐Nanometer Thick Gold Nanosheets as Highly Efficient Catalysts. Adv. Sci. 6 (21), 1900911. 10.1002/advs.201900911 PMC6839621 31728277

[B73] Yeh Y.-C. Creran B. Rotello V. M. (2012). Gold Nanoparticles: Preparation, Properties, and Applications in Bionanotechnology. Nanoscale 4 (6), 1871–1880. 10.1039/c1nr11188d 22076024 PMC4101904

[B74] Yi C. Liu D. Fong C.-C. Zhang J. Yang M. (2010). Gold Nanoparticles Promote Osteogenic Differentiation of Mesenchymal Stem Cells through P38 MAPK Pathway. ACS Nano 4 (11), 6439–6448. 10.1021/nn101373r 21028783

[B75] Yuan M. Wang Y. Qin Y. X. (2017). SPIO‐Au Core-Shell Nanoparticles for Promoting Osteogenic Differentiation of MC3T3‐E1 Cells: Concentration‐dependence Study. J. Biomed. Mater. Res. 105 (12), 3350–3359. 10.1002/jbm.a.36200 PMC5761339 28869707

[B76] Zeng L. Geng H. Gu W. Ma S. Qin Y. Xia S. (2019). Au Nanoparticles Attenuate RANKL-Induced Osteoclastogenesis by Suppressing Pre-Osteoclast Fusion. J. Nanosci Nanotechnol 19 (4), 2166–2173. 10.1166/jnn.2019.15764 30486961

[B77] Zhang D. Liu D. Zhang J. Fong C. Yang M. (2014). Gold Nanoparticles Stimulate Differentiation and Mineralization of Primary Osteoblasts through the ERK/MAPK Signaling Pathway. Mater. Sci. Eng. C 42, 70–77. 10.1016/j.msec.2014.04.042 25063094

[B78] Zhang X. Pan S. Song H. Guo W. Gu F. Yan C. (2021a). Photothermal Effect Enables Markedly Enhanced Oxygen Reduction and Evolution Activities for High-Performance Zn-Air Batteries. J. Mater. Chem. A. 10.1039/D1TA03652A

[B79] Zhang X. Pan S. Song H. Guo W. Zhao S. Chen G. (2021b). Polymer-Inorganic Thermoelectric Nanomaterials: Electrical Properties, Interfacial Chemistry Engineering, and Devices. Front. Chem. 9, 677821. 10.3389/fchem.2021.677821 33981678 PMC8107684

[B80] Zhang Y. Jin Y. He M. Zhou L. Xu T. Yuan R. (2018). Optical Properties of Bimetallic Au-Cu Nanocrystals Embedded in Glass. Mater. Res. Bull. 98, 94–102. 10.1016/j.materresbull.2017.10.009

[B81] Zhang Y. Jordan J. M. (2010). Epidemiology of Osteoarthritis. Clin. Geriatr. Med. 26 (3), 355–369. 10.1016/j.cger.2010.03.001 20699159 PMC2920533

[B82] Zhang Y. Kong N. Zhang Y. Yang W. Yan F. (2017a). Size-dependent Effects of Gold Nanoparticles on Osteogenic Differentiation of Human Periodontal Ligament Progenitor Cells. Theranostics 7 (5), 1214–1224. 10.7150/thno.17252 28435460 PMC5399588

[B83] Zhang Y. Zhang J. Zhang J. Lin S. Huang Y. Yuan R. (2017b). Intense Enhancement of Yellow Luminescent Carbon Dots Coupled with Gold Nanoparticles toward white LED. Dyes Pigm. 140, 122–130. 10.1016/j.dyepig.2017.01.043

[B84] Zhao F. Zhao Y. Liu Y. Chang X. Chen C. Zhao Y. (2011). Cellular Uptake, Intracellular Trafficking, and Cytotoxicity of Nanomaterials. Small 7 (10), 1322–1337. 10.1002/smll.201100001 21520409

[B85] Zhao M. Chen T. He B. Hu X. Huang J. Yi P. (2020). Photothermal Effect-Enhanced Photoelectrochemical Water Splitting of a BiVO4 Photoanode Modified with Dual-Functional Polyaniline. J. Mater. Chem. A. 8 (31), 15976–15983. 10.1039/d0ta03698f

[B86] Zhao X. Huang Q. Jin Y. (2015). Gold Nanorod Delivery of LSD1 siRNA Induces Human Mesenchymal Stem Cell Differentiation. Mater. Sci. Eng. C 54, 142–149. 10.1016/j.msec.2015.05.013 26046277

[B87] Zhao Y.-F. Zeng D.-L. Xia L.-G. Zhang S.-M. Xu L.-Y. Jiang X.-Q. (2013). Osteogenic Potential of Bone Marrow Stromal Cells Derived from Streptozotocin-Induced Diabetic Rats. Int. J. Mol. Med. 31 (3), 614–620. 10.3892/ijmm.2013.1227 23292283

[B88] Zhou J. Zhang Y. Li L. Fu H. Yang W. Yan F. (2018). Human β-defensin 3-combined Gold Nanoparticles for Enhancement of Osteogenic Differentiation of Human Periodontal Ligament Cells in Inflammatory Microenvironments. Int J. Nanomedicine. 13, 555–567. 10.2147/IJN.S150897 29416335 PMC5790078

[B89] Zhou Y. Millward-Sadler S. J. Lin H. Robinson H. Goldring M. Salter D. M. (2007). Evidence for JNK-dependent Up-Regulation of Proteoglycan Synthesis and for Activation of JNK1 Following Cyclical Mechanical Stimulation in a Human Chondrocyte Culture Model. Osteoarthritis and Cartilage 15 (8), 884–893. 10.1016/j.joca.2007.02.001 17408985

[B90] Zhu J. Lu N. Chen W. Kong L. Yang Y. Ma D. (2015). Influence of Au Nanoparticle Shape on Au@Cu2O Heterostructures. J. Nanomater. 2015, 389790. 10.1155/2015/389790

[B91] Zhu S. Zhu L. Yu J. Wang Y. Peng B. (2019). Anti-osteoclastogenic Effect of Epigallocatechin Gallate-Functionalized Gold Nanoparticles *In Vitro* and *In Vivo* . Int J. Nanomedicine. 14, 5017–5032. 10.2147/IJN.S204628 31371944 PMC6627179

